# Explaining the Atypical Reaction Profiles of Heme Enzymes with a Novel Mechanistic Hypothesis and Kinetic Treatment

**DOI:** 10.1371/journal.pone.0010601

**Published:** 2010-05-17

**Authors:** Kelath Murali Manoj, Arun Baburaj, Binoy Ephraim, Febin Pappachan, Pravitha Parapurathu Maviliparambathu, Umesh K. Vijayan, Sivaprasad Valiyaveettil Narayanan, Kalaiselvi Periasamy, Ebi Ashley George, Lazar T. Mathew

**Affiliations:** 1 Center for BioMedical Research, Vellore Institute of Technology University, Vellore, Tamilnadu, India; 2 Mala Education Trust (MET's) School of Engineering, Mala, Thrissur, India; University of Delhi, India

## Abstract

Many heme enzymes show remarkable versatility and atypical kinetics. The fungal extracellular enzyme chloroperoxidase (CPO) characterizes a variety of one and two electron redox reactions in the presence of hydroperoxides. A structural counterpart, found in mammalian microsomal cytochrome P450 (CYP), uses molecular oxygen plus NADPH for the oxidative metabolism (predominantly hydroxylation) of substrate in conjunction with a redox partner enzyme, cytochrome P450 reductase. In this study, we employ the two above-mentioned heme-thiolate proteins to probe the reaction kinetics and mechanism of heme enzymes. Hitherto, a substrate inhibition model based upon non-productive binding of substrate (two-site model) was used to account for the inhibition of reaction at higher substrate concentrations for the CYP reaction systems. Herein, the observation of substrate inhibition is shown for both peroxide and final substrate in CPO catalyzed peroxidations. Further, analogy is drawn in the “steady state kinetics” of CPO and CYP reaction systems. New experimental observations and analyses indicate that a scheme of competing reactions (involving primary product with enzyme or other reaction components/intermediates) is relevant in such complex reaction mixtures. The presence of non-selective reactive intermediate(s) affords alternate reaction routes at various substrate/product concentrations, thereby leading to a lowered detectable concentration of “the product of interest” in the reaction milieu. Occam's razor favors the new hypothesis. With the new hypothesis as foundation, a new biphasic treatment to analyze the kinetics is put forth. We also introduce a key concept of “substrate concentration at maximum observed rate”. The new treatment affords a more acceptable fit for observable experimental kinetic data of heme redox enzymes.

## Introduction

Hemoproteins serve multiple roles in the cellular biochemistry and as a result, they are one of the well-studied proteins. Structure-function similarities as well as disparities can be drawn between the two heme proteins, chloroperoxidase (CPO) and cytochrome P450 (CYP). The former is a highly stable glycosylated extracellular acidic protein with a constrained and polar active site [1]. The latter is a relatively sensitive microsomal membranous protein showing little post translational modifications, but with a larger hydrophobic active site [2], [3]. Though both are ∼45 KD mass and ∼45 Å in dimensions, they bear only ∼25% sequence similarities. The most important common structural element is the proximal thiolate ligand, bound to the central iron of heme (protoporphyrin IX). The formal charge on iron at the resting state is 3^+^and the spin state may change based on the distal ligand and microenvironment. Both these enzymes are well-studied enzyme systems, characteristically marked for their versatility in the number of reactions they can catalyze [4]–[7]. Both CPO and CYPs are known for a relative lack of specificity in substrate preferences. Chloroperoxidase is a classical peroxygenase carrying out its oxidations with hydroperoxide as the ancillary activator [8]. CYP is a typical monooxygenase which requires a ternary mixture of molecular oxygen, yet another enzyme called cytochrome P450 reductase (CPR) and redox equivalents from NADPH [9].

**Figure 1:**
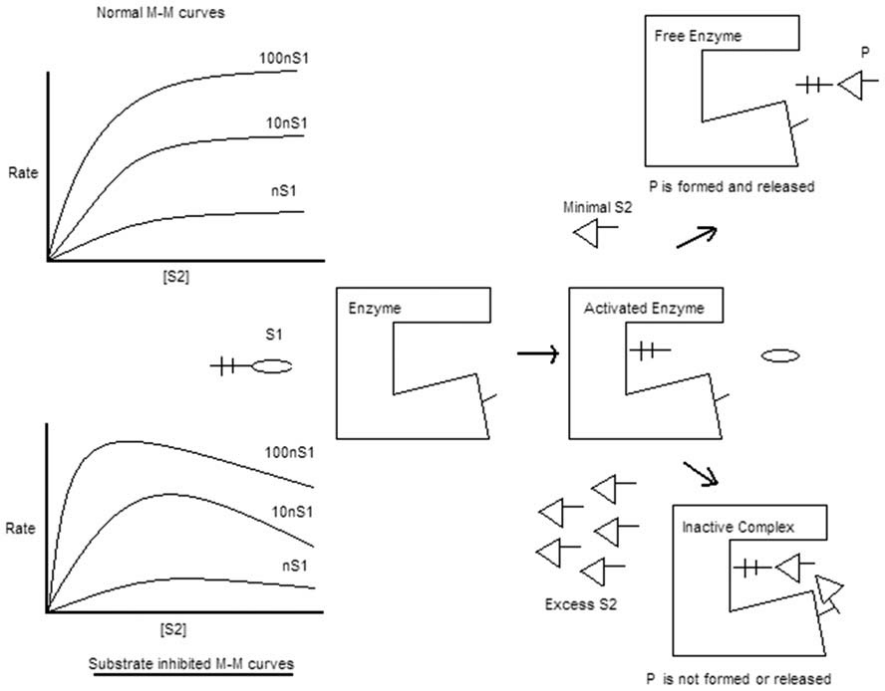
The usual or unusual kinetic profiles and their mechanistic explanations hitherto available are shown. The profile at top-left shows kinetics via a simple Michaelis-Menten bisubstrate mechanism, corresponding to a mechanistic scheme shown at top-right. At bottom-left is the substrate inhibited kinetics profile, which is explained mechanistically at the bottom-right.

Our recent explorations with chloroperoxidase catalyzed peroxidations had afforded some interesting and unexpected results. To account for these observations, we had reasoned out that some of the enzyme-substrate (final acceptor) interactions might not go through the typical enzyme-substrate binding [10] at a unique active site of the enzyme. As a result, they may not be defined by the classical Michaelis-Menten kinetics. While pursuing the newly discovered angles on the single electron peroxidations of CPO, we found some analogies with the two-electron CYP catalyzed oxygen insertion reactions, pertaining to the “inhibition of reaction” at higher substrate concentrations. Approximately 20% of the known enzymes have shown some form of substrate inhibition. Hitherto, such atypical kinetic behavior was attributed to a mechanistic scheme involving alternate substrate binding site(s) near the CYP enzyme's active site[11]–[14], which is a two-site binding model, as shown in Figure 1.

We present evidence and argue herein that it is highly unlikely that both CPO and the CYP family of enzymes (as exemplified by CYPs 2C9, 2E1 & 1A2) possess multiple binding sites for their diverse array of substrates. To explain for the ‘substrate inhibition’, we propose an alternative hypothesis which involves enzyme-free reactions of the substrates, product(s) and transient intermediate(s). We also present an alternative model for fitting the data obtained, to give meaningful indices of the reaction system.

## Results

### CPO mediated peroxidations of ABTS, TMPD and Pyrogallol

To derive an overview of the steady-state reaction profiles, the substrate molecules' concentrations were varied over three decades. Kinetic data obtained for the production of stable cation radicals and condensed products were noted within the first few tens of seconds of reaction are summarized in Table 1.

**Table 1:** Analyses of CPO catalyzed peroxidations by Belanger fits.

Substrate	Reaction Setup	K_M_ (mM)		V_max_ (s^−1^)		K_IS_ (mM)		*R^2^*
***Peroxide***	25 µM ABTS	0.11±0.04	0–0.24	17.5±3.4	6.8–28.3	0.67±0.21	0–1.3	0.991
	0.25 mM ABTS	0.11±0.15	0–0.58	102±71	0–326	0.39±0.39	0–1.6	0.953
	2.5 mM ABTS	0.14±0.08	0–0.40	202±45	60–344	2.3±1.1	0–5.8	0.930
	25 µM TMPD	0.01±0.03	0–0.08	44.5±6.8	29.2–59.8	86.6±234	0–616	0.014
	0.25 mM TMPD	0.12±0.06	0–0.26	421±80	240–601	1.9±0.75	0.2–3.6	0.835
	2.5 mM TMPD	0.46±0.2	0.01–0.92	2014±378	1158–2869	5.5±2.2	0.5–10.6	0.879
	25 µM Pyrogallol	0.08±0.05	0–0.19	7±1	4.8–9.3	15.1±9.8	0–37.4	0.461
	0.25 mM Pyrogallol	0.08±0.02	0.04–0.13	59.2±2.9	52.7–65.8	13.6±2.9	7.1–20	0.879
	2.5 mM Pyrogallol	1.05±0.12	0.79–1.33	509±28	446–572	29.1±7	13.3–45	0.995
***ABTS***	0.1 mM Peroxide	0.37±0.03	0.30–0.45	44.6±1.3	41.6–47.5	*Nd*		0.994
	1 mM Peroxide	0.91±0.14	0.59–1.22	76.2±5.3	64.5–87.9	*Nd*		0.989
	10 mM Peroxide	1.96±0.21	1.48–2.43	23±1.5	19.8–26.2	*Nd*		0.997
***TMPD***	0.1 mM Peroxide	0.58±0.18	0.09–1.08	188±22	126–250	*Nd*		0.973
	1 mM Peroxide	3.2±1	0.41–6.03	1366±28	566–2166	*Nd*		0.995
	10 mM Peroxide	9.1±4.2	0.0–20.9	2099±820	0–4377	*Nd*		0.998
***Pyrogallol***	0.1 mM Peroxide	0.13±0.05	0.03–0.23	36.3±3.2	29.1–43.5	*Nd*		0.908
	1 mM Peroxide	1.43±0.31	0.75–2.12	312±35	235–389	*Nd*		0.985
	10 mM Peroxide	13.4±3.6	5.43–21.5	2210±523	1045–3375	*Nd*		0.999
	2 mM Peroxide*	9.05±2.8	3.2–14.9	1846±212	1400–2292	168±39	84–251	0.920

Kinetic constants shown in the first three columns are average of duplicate experiments (with standard deviation) and 95% confidence intervals respectively.

*pH 3, CPO = 1.8 nM, 25°C.

**Figure 2 pone-0010601-g002:**
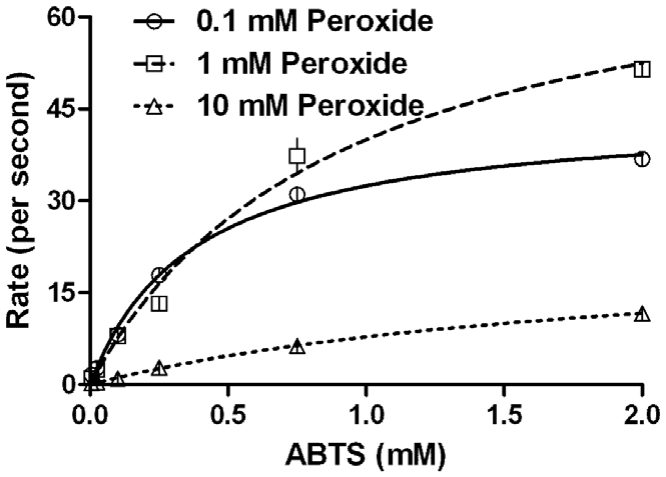
Kinetics of CPO catalyzed peroxidation of ABTS obtained by varying the ABTS concentration, at constant peroxide. Initial conditions- pH 3.5, 100 mM phosphate buffer, 30°C, [CPO] = 20 nM.

**Figure 3 pone-0010601-g003:**
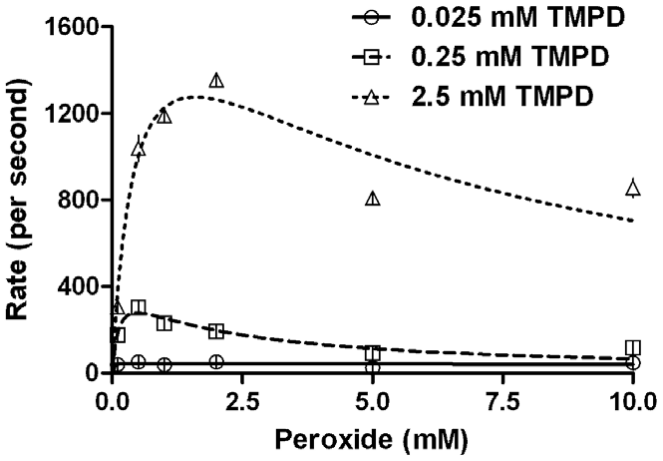
Kinetics of CPO catalyzed peroxidation of TMPD obtained by varying the peroxide concentration, at constant peroxidative substrate (TMPD). Initial conditions- pH 3.5, 100 mM phosphate buffer, 30°C, [CPO] = 20 nM.

For all reactions studied, increasing the final peroxidative substrates' concentration (at an initial constant peroxide concentration) generally gave an increase in the reaction rates for the sub-millimolar reaction range studied (Figure 2 is a salient example for ABTS, other data are shown in Table 1), which could be fit to non-linear regression of Michaelis-Menten equation, with R^2^ values above 0.9. However, the data could not be transformed to fit the popular linearized models exemplified by Lineweaver-Burk or Eadie-Hofstee (results not shown). Also, incrementing the first substrate concentration by decades did not give corresponding levels of increase in second substrate conversion, as expected in Figure 1 (left side). The reaction profiles obtained by varying peroxide concentrations at constant peroxidative substrate concentration gave mixed trends (Figure 3 & Table 1 show the profile for TMPD). The increase in peroxide concentrations gave an apparent inhibition at higher concentrations, the extent of which varied with different peroxidative substrates. Again, none of these profiles could be fit to a non-linear substrate inhibition model within an extension of the Michaelis-Menten paradigm (as exemplified by the Belanger fit [15]) to give global and reproducible constants with accuracy and precision (Table 1).

**Figure 4 pone-0010601-g004:**
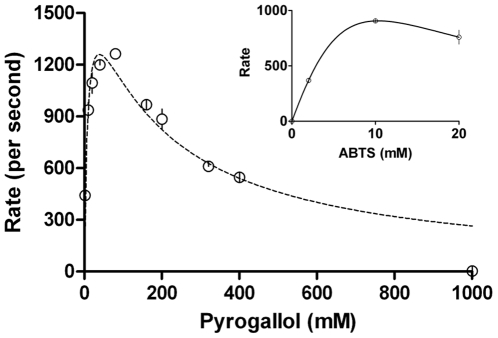
Kinetics of CPO catalyzed peroxidation of Pyrogallol (and ABTS in the inset) obtained by varying ABTS concentration to supramillimolar levels, at constant peroxide. Initial conditions- pH 3, 100 mM phosphate buffer, 25°C, [CPO] = 2 nM, peroxide  = 2 mM.

Figure 4 shows that a substrate like pyrogallol, which showed lower inhibitions with peroxide increments, also exhibited lowered product (purpurogallin) formation at supra-millimolar concentrations of pyrogallol. The inhibition was also seen for an active site excluded substrate like ABTS (inset of Figure 4). When the two sets of data (rates obtained by varying peroxide at constant final peroxidative substrate versus rates obtained by varying final peroxidative substrate at constant peroxide) were compared, it could be seen that increase in peroxide (at constant peroxidative substrate) had a greater effect on lowering rate. In most cases, the Belanger fit curves did not justifiably cover the data points at higher substrate concentrations (data not shown).

### CYP2E1 reactions with pNP

**Figure 5 pone-0010601-g005:**
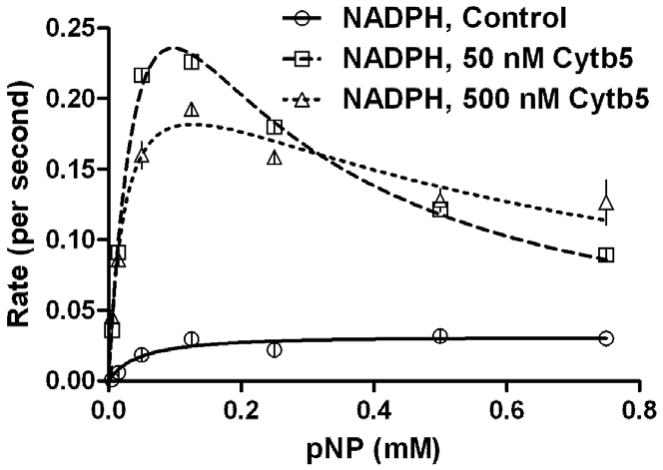
Steady state kinetics of reconstituted CYP2E1 enzymatic system mediated conversion of pNP with NADPH added directly, as a function of incorporated Cytb_5_. Experimental details are given in methods section.

**Figure 6:**
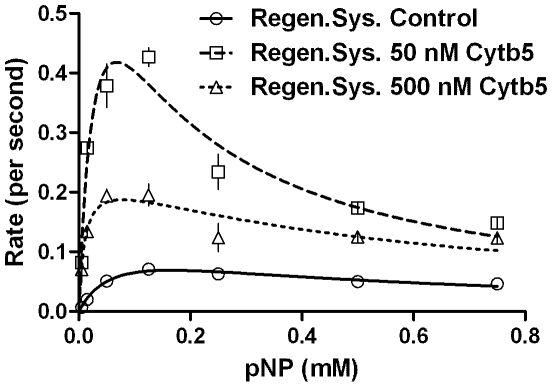
State kinetics of reconstituted CYP2E1 enzymatic system mediated conversion of pNP with NADPH regeneration system, as a function of incorporated Cytb_5_. Experimental details are given in methods section.

**Figure 7:**
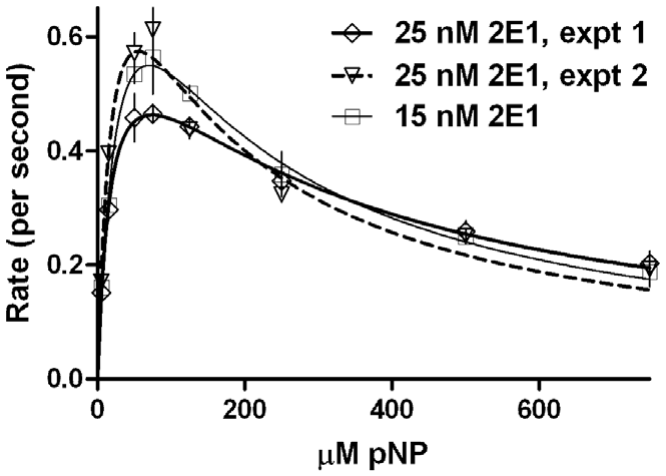
State kinetics of reconstituted CYP2E1 enzymatic system mediated conversion of pNP, checked for reproducibility and precision. Experimental details are given in methods section.

**Figure 8:**
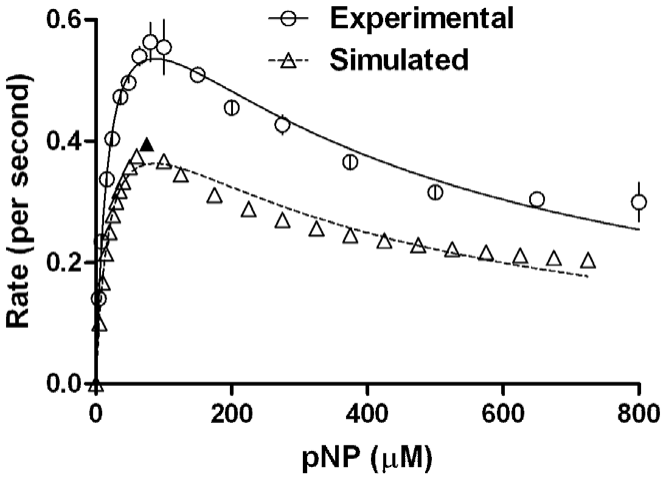
CYP2E1 kinetics, probed in detail using experimental data and theoretical simulation. Experimental details are given in methods section.

Figures 5 & 6 and Table 2 depict the result when substrate effect was probed in two reaction systems- (1) employing NADPH regeneration system with enzymes to remove reduced oxygen species (ROS) and (2) directly with NADPH and without enzymes included to remove ROS. In both these reaction systems, employment of moderate amounts of Cyt*b*_5_ enhanced the product formation at lower substrate concentrations when compared to the controls. Notably, the first setup gave much higher yields of product in comparison to the second. However, incorporation of excess Cyt*b*_5_ gave similar product formations (especially at higher substrate concentrations ranges) for both setups. The effect of CYP concentration and reproducibility was probed and the results are shown in Figure 7 & Table 2. Clearly, the magnitude of K_IS_ varied from one experiment to another significantly. The reaction profile was charted in detail by taking a 16 point curve (Figure 8), which showed that the descending part of the Belanger fit (after inflexion) significantly deviated from the experimental points. A theoretical simulation using the double hyperbolic combination showed a similar profile as the experimentally determined points (Figure 8).

**Table 2:** Analyses of CYP catalyzed peroxidations by Belanger fits.

Reaction	Parameters	K_M_ (µM)		V_max_ (s^−1^) or (min-1)@		K_IS_ (mM)		*R^2^*
***CYP2E1 + pNP***	*NADPH, control*	48±38	0–155	0.034±0.01	0.005–0.06	9.48±47.9	0–142	0.912
	*NADPH, x Cytb_5_*	73±17	25–120	0.59±0.09	0.337–0.84	0.13±0.03	0.05–0.2	0.993
	*NADPH, 10x Cytb_5_*	27±8	5–48	0.26±0.03	0.171–0.34	0.609±0.2	0.08–1.1	0.963
	*Regen.Sys., control*	72±23	9–135	0.13±0.02	0.067–0.19	0.384±0.1	0.04–0.7	0.983
	*Regen.Sys., x Cytb_5_*	35±21	0–93	0.85±0.3	0.03–1.67	0.13±0.07	0–0.34	0.926
	*Regen.Sys., 10x Cytb_5_*	11±6	0–27	0.24±0.04	0.12–0.36	0.56±0.28	0–1.33	0.818
	25 nM 2E1, Expt 1	18.5±2.1	14–23	0.7±0.03	0.63–0.76	0.295±0.03	0.23–0.4	0.982
	25 nM 2E1, Expt 2	25.8±6.5	9.9–38	1.07±0.15	0.744–1.39	0.129±0.03	0.06–0.2	0.938
	15 nM 2E1	33.2±6.2	20–47	1.07±0.11	0.842–1.3	0.148±0.03	0.09–0.2	0.973
	25 nM, 16 points	19±1.93	15–23	0.77±0.03	0.71–0.82	0.399±0.04	0.32–0.5	0.963
	Simulated	20.6±2.6	15–26	0.536±0.03	0.48–0.59	0.363±0.04	0.3–0.44	0.970
***CYP2C9 + Diclof***	X CYP2C9	2±0.8	0.2–3.8	0.129±0.01	0.097–0.16	0.255±0.11	0.01–0.5	0.673
	2X CYP2C9	2.6±0.9	0.6–4.6	0.148±0.02	0.12–0.18	0.15±0.05	0.04–0.3	0.780
***CYP1A2+ 7EFC^@^***	5 minutes	∼0	∼0	19.98±2.81	13.9–26	23.2±9.32	3.1–43.4	0.822
	10 minutes	115±219	0–588	39.89±7.17	24.4–55.4	18.9±8.9	0.0–38.1	0.782
	15 minutes	477±376	0–1290	64.88±14.4	33.8–95.9	13.4±6.5	0.0–27.5	0.787
	Averaged (0–15 min.)	2988±2544	0–9529	9.29±5.19	0–22.6	4.9±3.9	0.0–14.9	0.915

Kinetic constants shown in the first three columns are average of duplicate experiments (with standard deviation) and 95% confidence intervals respectively.

### CYP2C9 reactions with diclofenac

**Figure 9:**
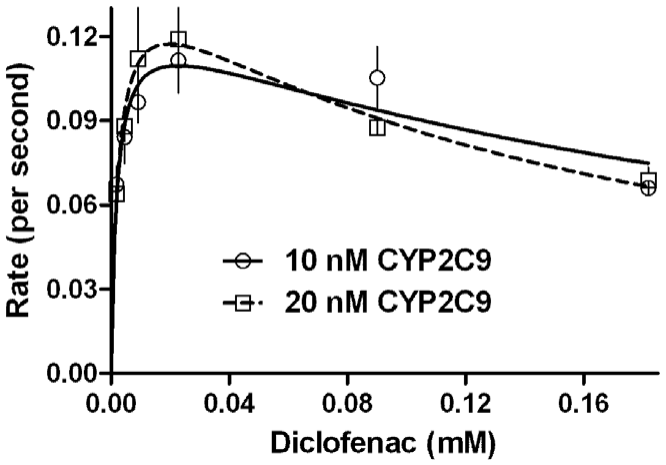
CYP2C9 baculosomes show lowering of specific hydroxylation of diclofenac upon increasing substrate concentration. A final concentration of 1 mM NADPH was used. 5 and 10 µl of the commercial enzyme preparation was added to 1 ml of the reaction mixture.

**Figure 10:**
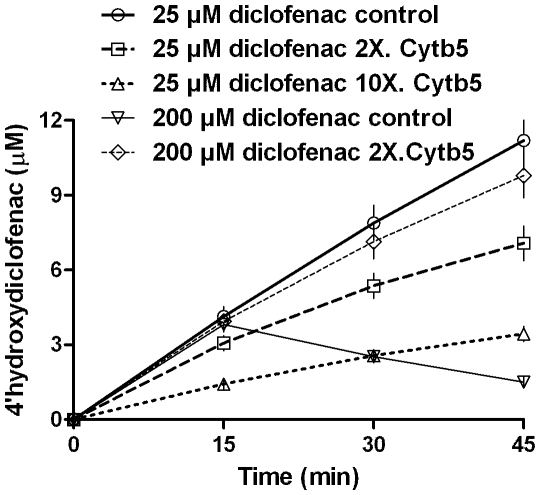
Reconstituted CYP2C9 system shows depletion of specific hydroxylated product over time. Initial concentrations are ∼0.08 uM of CYP2C9  =  CPR, 0.04 to 0.8 uM Cyt*b*_5_, 1 mM NADPH. Other details are mentioned in the experimental section.

Figure 9 shows the 4′hydroxylated product obtained for diclofenac's reaction with two concentrations of CYP2C9. The CYP concentration was shown to have pronounced effect on the value of constants (quite like the results with CYP2E1) and the “substrate inhibition” was more pronounced at higher enzyme concentration. When a similar reaction setup was probed with respect to product formation at various time intervals (Figure 10), it was noted that the formed product disappeared for higher substrate concentration. Incorporation of Cyt*b*_5_ lowered the product formation at low substrate concentrations but showed a positive effect on the yield of product at higher substrate concentrations.

### CYP1A2 reactions with 7-EFC

**Figure 11:**
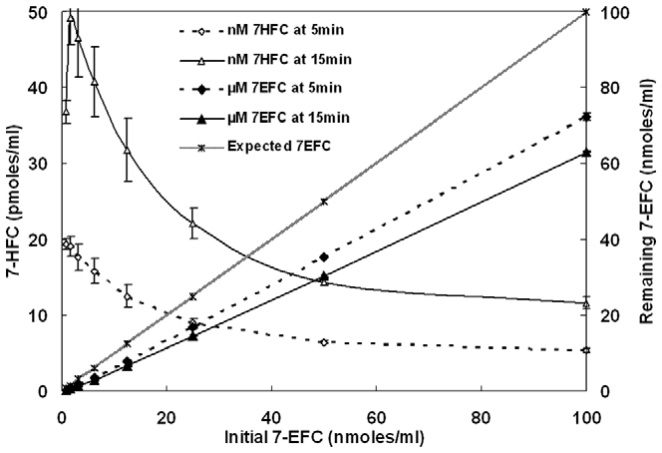
Reconstituted CYP1A2 system shows that the specific hydroxylated product formed does not account for the total substrate converted in the reaction. Initial concentrations were [CPR] = 200 nM, [CYP1A2] = 100 nM, [DLPC] = 20 µM, [NADPH] = 500 µM & varying [7EFC] stocks in DMSO were added at a final concentration of 1% of the cosolvent.

In these reactions, quantification of both the expected product (the specifically de-ethylated substrate) and the substrate was carried out after incubation for specific times. The results are shown in Figure 11. It could be seen that the product inhibition occurred at much lower substrate concentrations. Also, the amount of specific product formed did not account for the amount of substrate depleted. Extra peaks were seen in the chromatograms of these samples (results not shown) indicating the formation of new products. These unidentified new products could account for the disappearance of excess substrate (the substrate missing and not accounted for by the formation of the expected product of 7-hydroxyfluorocoumarin).

## Discussion

Some workers in the past have probed thiolate-peroxidases as a mechanistic model for CYP reactions, citing analogies and differences in their modes of action [16]–[20]. Their arguments relate to the details of the nature of the catalytic enzymatic species and the precise mechanistic chemistry involved in the reaction of the catalytic species with the substrate. Such discussions do not dictate the context of the present work. The aspect of mechanistic process probed here is if the ‘spatio-temporal’ idea of obstruction of the active site by higher concentration of substrates (or an allosteric modulation by excess substrate at an alternative enzyme binding site), as advocated erstwhile, and as is depicted in Figure 1, provides an adequate explanation to the ‘substrate inhibition’ kinetics.

**Figure 12:**
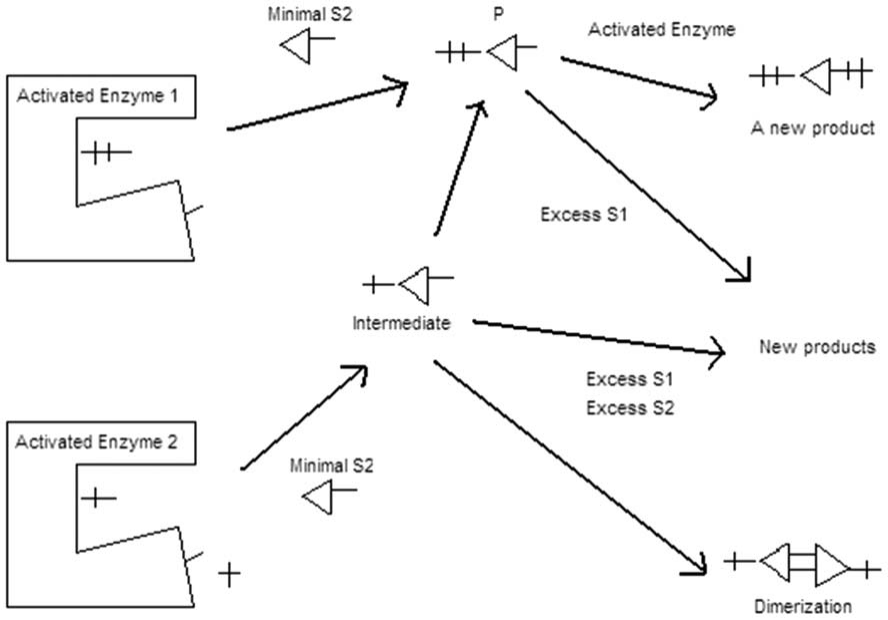
The newly proposed mechanistic possibilities in reactions catalyzed by hemoproteins are shown. At the left are two-electron (top) and one-electron (bottom) oxidized enzyme intermediates, giving rise to two-electron (top) and one-electron (lower) oxidized product or intermediates in the center. The final products that could be formed are shown to the right.

The kinetic profiles obtained in this work indicate that the single electron abstractions (which have been proven to not necessarily be finally catalyzed at a unique catalytic site by CPO [10]) show a lowered yield of product upon increasing any one of its substrates. The data from this work indicates that lowering of product yields at high substrate concentrations may not be owing to a process involving interaction of the substrate and the enzyme alone, for both CPO and CYP. Evidence presented in the reactions with CYPs show that these observations could very well be owing to other factors, as is shown in Figure 12. The mechanistic route could involve:

1. A transient intermediate (either an activated enzyme or substrate species) could react with excess substrate to give an alternate product, thereby lowering the product by a competitive process. To validate this argument- (a) Schlichting *et al.* have already demonstrated the existence of one and two electron activated CYP species [21]. Also, Guengerich *et al.* had earlier advocated a one-electron process mechanistic scheme for explaining the observations in some CYP reactions [17]. (b) Superoxide has been proposed as an essential mechanistic component (by one of us [22]) and demonstrated to exist in reaction milieu by many workers as a deleterious side-product [9]. It could react very feasibly with the substrate (which has been demonstrated earlier by one of us [22]) and reaction product on its own merit. Such a reaction would be anticipated to be a function of the substrate concentration, which could explain the lowered yield of specific product at higher substrate concentration.
OR/AND
2. The product formed could get further converted by the enzyme or yet, react with another reaction intermediate/component present in the milieu, thereby lowering the value of estimated product in the reaction setup. This inference has been amply supported by data from this study.

In several CPO catalyzed peroxidations, the colored peroxidation product formed was depleted in due course of the reaction on its own or/and by the addition of excess of the peroxidative substrate or peroxide itself (results not shown). This makes perfect chemical sense because these reaction components are non-specific redox sensitive molecules. CPO catalyzed chlorination reaction milieu has already been shown to possess such chemistry where peroxide serves both as activator and scavenger of reactive intermediate(s) [23]. Earlier, Cassella *et al* had shown that the oxidation of phenolics by CPO also exhibited a lowered product formation at higher peroxidative substrate concentration [24]. We found similar observation for various peroxidative substrates above 10 mM concentrations in our investigations (where solubility permitted, results not shown).

All these observations in steady-state kinetics of fungal CPO and microsomal CYPs indicate an analogy in the chemical reaction phenomena. It is generally understood that both fungal CPO and microsomal CYPs catalyze via a bi-substrate route. The first process in both enzymes is assumed to involve the oxygen donor species binding at the heme and the second involves the final electron donor/acceptor substrate interaction. We know that the original activator in the CPO reaction is the redox sensitive peroxide and if we could consider superoxide as an activator for CYPs (as mooted earlier by one of us [22]), then the analogy would be explained even better.

Incorporation of Cyt*b*_5_ in CYP reaction milieu (as a one-electron redox sensitive hydrophobic macromolecule) showed clear indications that single electron processes have direct relevance to the product formation and distribution in the milieu. Also, earlier interpretations were that Cyt*b*_5_ serves as a redox relay between CYPs and reductase, mediating the process via a protein-protein complexation [25], [26]. If that were to be the case, increase in Cyt*b*_5_ should only increase the product yield. This is not observed and therefore, the erstwhile hypothesis that “Cyt*b*_5_ is a protein-protein redox relay between CYP and CPR” is questionable. On the other hand, if we assume Cyt*b*_5_ to be a non-selective one-electron transient scavenger cum cycler (that affects the equilibriums involving single and two electron processes at the lipid interface) in a non-specific redox reaction setup involving superoxide, we could efficiently account for lowering or enhancement of rates in various compositions. This is especially evident as excess Cyt*b*_5_ serves well in both CYP2E1 and CYP2C9 reactions at higher substrate concentrations, under diverse conditions.

The concentration range of substrate at which the maximal rates were observed (following which increase in substrate concentrations showed a lowering in product yield) for the three CYPs ranged around 5, 25 and 125 µM for CYP1A2: 7-EFC, CYP2C9: diclofenac and CYP2E1: pNP respectively. This observation is in accordance with the chemical reactivity of the substrates. That is - the electron withdrawing nitro group and an adjacent sterically hindering hydroxyl makes pNP molecules' reaction centre to be less efficient. In comparison, the non-hindered ethoxy group's oxygen moiety's lone pair (which is conjugated to the π electron cloud in the coumarin derivative and also connected to an electron donating ethyl group) would be the most reactive and easily accessible center among the three substrates tested. In case of the peroxidation reaction with CPO, the active-site-excluded ABTS [10] (which has better electron density and donating ability) shows inhibition at lower substrate concentrations than the smaller TMPD (for a given peroxide concentration). This is yet another consideration which indicates that the “substrate inhibition” is not connected to a blocking of the active site.

Surely, one cannot argue that there are no multiple binding sites for the various substrates on CYP and CPO. However, it is very difficult to imagine that enzymes like CPO and CYP family have multiple binding sites for their diverse array of both their final substrates and initial activator (peroxide). As per the data reported in this study, the non-specific reaction network put forward in Figure 12 appears more probable. Therefore, at this juncture, we express a note of caution towards the data and interpretations published in a recent article [27]. The authors therein have reported kinetic and equilibrium constants for effector site modulations (alternate term for substrate inhibitions) in the activity of CYP2E1-pNP reaction mixtures. We have exhaustively worked on the same system and ascertained that it is impossible to derive any reproducible and meaningful kinetic constants for steady state kinetics using experimental data by non-linear regression analyses with available kinetic treatments or software. As can be evident from a visual examination of Figure 2 from their communication, the theoretical fits do not do justice to the experimental data. Specifically, the rates at the point of inflexion (where maximum activities are obtained) and higher substrate concentrations (the region that the hypothesis supposedly accounts for!) clearly go out of the fit. These outliers are real and not just instances of experimental error, as hitherto considered. With much lesser standard deviations of individual points, better R^2^ values than the data reported therein and with more number of points spread through the curve, the value of K_IS_ obtained with Dynafit plots in our work ranged from ∼100 µM to ∼10 mM in simple control reactions that did not include any inhibitor. When molecules (inhibitors) like 4-methylpyrazole and isoquinoline were added (in our study, results not shown), the complexities for determining K_I_ and K_IS_ only increased manifold. The fits then pointed to an uncompetitive or noncompetitive model, at times a mixed model and in some instances, the fitted curve could hardly be called a fit! Therefore, it is our view that attempts to draw any interpretations from such poor fits and inappropriate hypothesis would only lead one to erroneous conclusions. Merely, getting an R^2^ value above 0.9 is no indication that the hitherto proposed model has fidelity to the equation or mechanism. An exhaustive study of the two tables presented herewith (along with the respective figures) would convince any careful reader of the above statement.

The simulation of kinetics (and fitting thereof) could be achieved better using a biphasic treatment, with the incorporation of new meaningful constants. The fitting (for rate Y versus substrate concentration X) could be done with the following simple algorithm, using two intermediate variables (Y1 & Y2) and six true variables-
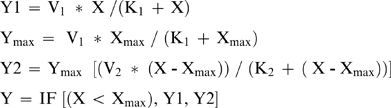



**Figure 13:**
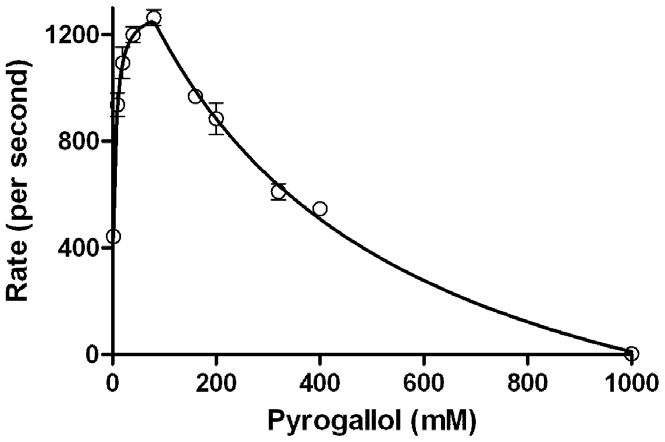
The new biphasic fit is plotted for CPO mediated peroxidation profile for conversion of pyrogallol from **Figure 4** **.**

**Figure 14:**
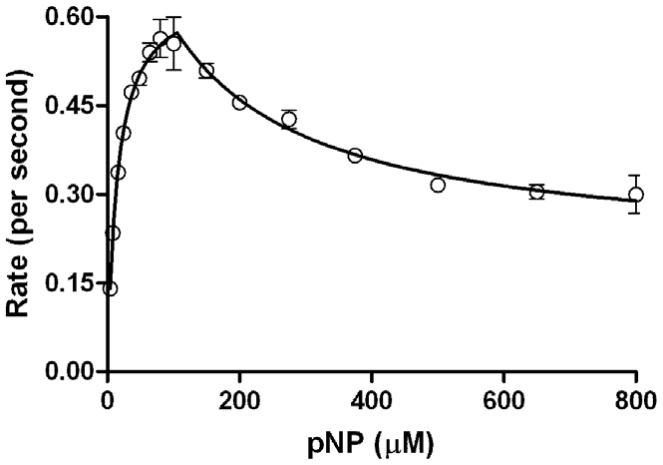
The new biphasic fit is plotted for CYP2E1 mediated hydroxylation profile for conversion of pNP from **Figure 8**.

The last expression is a logic statement in the algorithm used to fit data serving to demarcate the two hyperbolic phases with (X_max_, Y_max_) as the node. Of the six true variables, V_1_ & K_1_ are the theoretical constants for ascending part & V_2_ & K_2_ are the theoretical constants for the descending part. The practically observable and ascertainable Y_max_ (the maximum observable rate) and X_max_ (the concentration of substrate at which maximum rate is observed) values are the new concepts introduced here. The latter values could convey more meaningful information of a given reaction system in which ‘substrate inhibition’ is expected or observed. The re-analyses of experimental data for CPO's reaction profile with pyrogallol (Figure 4) with the new fitting method gave an R^2^ value  = 0.994. The result is shown in Figure 13. When data from Figure 8 (for CYP2E1's experimental reaction profile with pNP) was refitted using the new method, an R^2^ value  = 0.983 was derived with the points falling much better onto the fitted curve (results are shown in Figure 14). These show that the novel biphasic treatment gives a far more satisfactory fit, as is determined with a simple visual verification. Other details of fitting and data obtained thereof shall be discussed in a forthcoming communication.

To conclude, the hemoprotein reaction milieu with two substrates (or more) involves several competing reactions. The exact nature of the product(s) formed and their distribution thereof would be dependant on the nature of enzyme and substrates, reaction conditions and reactant concentrations. Algorithms need to be developed to predict the probabilistic course of such a reaction, given a hemoprotein reaction mixture. These efforts would have far-reaching impact in our understanding of cellular and physiological processes involving these maverick enzymes.

## Materials and Methods

The chemicals and reagents used were of analytical grade, purchased from Sigma, Lancaster (USA) and SD Fine Chemicals (India). For procedural details on CPO, please refer to our recent publication [10]. The source of CYPs was either a commercial baculosome preparation from Invitrogen (PanVera) or reconstituted systems made in lab. For the production, purification and processing of CYP and associated proteins, please refer to an earlier publication [28].

### General conditions

CPO reactions were carried out in open with ∼20 nM CPO at ∼30±2°C, 100 mM phosphate buffer (Figures 1 through [Fig pone-0010601-g002] [Fig pone-0010601-g003] [Fig pone-0010601-g004] [Fig pone-0010601-g005] 6). The selection of pH value employed simple logic to optimize between autocatalytic rates and enzyme-catalyzed rates. Pyrogallol reactions were incubated at pH 5, ABTS [2,2′-azino-bis(3-ethylbenzthiazoline-6-sulphonic acid)] reactions were incubated at pH 3.5 and tetramethyl phenylene diamine (TMPD) reactions were incubated at pH 2.75. All CYP reaction incubations were done in aerated open vials at 37±1°C in 100 mM phosphate buffer, pH 7.4. Unless otherwise mentioned, reconstituted systems had 10 µg/ml of 0.2 µm vesicles of dilauryl phosphatidylcholine (DLPC, Avanti Lipids). Besides the general procedures mentioned below, specific components' concentrations and other details are mentioned in the legends to the corresponding figures.

### Reactions with CYP2E1

The reactions contained 20 µM DLPC, 15 nM CYP2E1, 60 nM CPR, 0/50/500 nM cytochrome *b*_5_ (Cyt*b*_5_) and the appropriate concentrations of para-nitrophenol (pNP). 200 µl of the regeneration system reaction mixture had - 0.3 µl of 2 M MgCl_2_, 2.4 µl of 5 µg/µl of superoxide dismutase, 0.6 µl of 1000 Units/µl catalase, 0.6 µl of 1 Unit/µl glucose-6-phosphate dehydrogenase & 60 µl of glucose-6-phosphate. The reactions were initiated with the addition of 500 µM NADP^+^ in the regeneration system reaction and 500 µM NADPH in another set. The HPLC protocol for quantification of product of para-nitrocatechol (pNC) was similar to a procedure reported recently [27]. Aliquots of the reaction mixture were taken at 6, 12 and 18 minutes and the rate of production of pNC was calculated by the slope of linear plot obtained.

### Reactions with CYP2C9

In another reaction involving reconstituted enzymes, 80 nM CYP + 80 nM CPR mixture was used. Cyt*b*5 was in the range of 40 to 800 nM (Figure 13). Termination of reaction for product quantification was achieved by adding 200 µl of a cold solvent mixture (94% acetonitrile + 6% acetic acid, containing *tert*-butyl phenol as the internal standard) to 500 µl of sample. Conversion of diclofenac (Diclof) was determined using Alltima C_18_ 5 µ HPLC column (150 mm×3.2 mm) from Alltech. The solvent system of 70% (of 30% acetonitrile in water with 1 mM perchloric acid) and 30% methanol was pumped at 0.8 ml/min. Chromatography was monitored with a UV detector at 267 and 275 nm. Elution times and areas of standard samples of diclofenac and its 3′, 4′ & 5′ hydroxylated derivatives were used for detecting and quantifying the products. Under the conditions employed, 4′hydroxydiclofenac and diclofenac eluted at ∼6 and 7.5 minutes respectively.

### Reactions with CYP1A2

At designated incubation intervals (5, 10 & 15 minutes), 75 µl of the reaction sample was taken and quenched with 25 µl of acetonitrile containing the internal standard and centrifuged. An appropriate amount (usually 40 µl of the supernatant) was injected for HPLC. The product formed was estimated from the slope of line formed by fitting a standard linear plot. For the quantification of 7-hydroxy trifluoromethylcoumarin (7-HFC), the de-ethylated product of CYP1A2 oxidation of 7-ethoxy trifluoromethylcoumarin (7-EFC), a Symmetry C_18_ 3.5 µm column from Waters was employed. A binary solvent system- solvent A = 75% of 0.1% trifluoroacetic acid in water & solvent B  =  acetonitrile were taken with a gradient method. The details are 0 min  = 1 ml/min, 75% A + 25% B; 4 min = 1.5 ml/min, 75% A + 25% B; 9 min = 1.5 ml/min, 0% A + 100% B; 11 min = 1.5 ml/min, 75% A + 25% B & 12 min = 1 ml/min, 75% A + 25%B. A fluorescence detector with excitation and emission wavelengths set at 385 nm and 500 nm respectively was used to quantify the reactants and products. The UV absorption profiles of products were also followed in certain reactions. The analytes 7-hydroxycoumarin (an internal standard taken at 20 µM), 7-HFC and 7-EFC eluted at ∼2.6, 8.2 and 9.6 minutes respectively.

### Fitting and analyses of kinetics data

Non-linear (and linear) regression analyses of enzyme kinetics were carried out using Dynafit and GraphPad Prism software for uncompetitive substrate inhibition (Belanger fit):
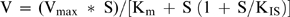



The constraints were only experimental values were taken (0,0 excluded) and V_max_, K_M_ and K_IS_>0. Both Dynafit and Prism outputs gave similar values of constants for all reactions studied herein and therefore, only the latter is reported. For theoretical simulation (as shown by the triangles in Figure 9), the data were derived using a fusion of two equations, in a biphasic treatment. The ascending points [until the blackened point- (75, 0.4) in Figure 9] were derived using the ascending hyperbola equation:
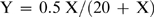



The descending points [from the blackened triangle point- (75, 0.4)] were derived using the descending hyperbola equation:
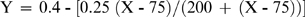



## References

[1] Sundaramoorthy M, Terner J, Poulos TL (1995). The crystal structure of chloroperoxidase: A heme peroxidase-cytochrome P450 functional hybrid.. Structure.

[2] Williams PA, Cosme J, Vinkovic DM, Ward A, Angove HC (2004). Crystal structures of human cytochrome P450 3A4 bound to metyrapone and progesterone.. Science.

[3] Wester RM, Yano JK, Schoch GA, Yang C, Griffin KJ (2004). The structure of human cytochrome P450 2C9 complexed with flurbiprofen at 2 Å resolution.. J Biol Chem.

[4] Porter TD, Coon MJ (1991). Cytochrome P450: Multiplicity of isoforms, substrate and catalytic and regulatory mechanisms.. J Biol Chem.

[5] Ortez de Montellano PR, Choe YS, Depillis G, Catalano CE (1987). Structure-mechanism relationships in hemoproteins.. J Biol Chem.

[6] Libby RD, Thomas JA, Kaiser LW, Hager LP (1981). Chloroperoxidase halogenation reactions.. J Biol Chem.

[7] Guengerich FP (1991). Reactions and significance of cytochrome P450 enzymes.. J Biol Chem.

[8] Manoj KM, Hager LP (2002). The catalytic utility and versatility of chloroperoxidase.. Rec Res Dev Org Chem.

[9] Ortiz de Montellano PR (1995). Cytochrome P450: Structure, mechanism and biochemistry..

[10] Manoj KM, Hager LP (2008). Chloroperoxidase, a Janus enzyme.. Biochemistry.

[11] LinY, Tang C, Mei Q, Sandig G, Rodrigues DA (2001). Substrate inhibition kinetics for cytochrome P450 catalyzed reactions.. Drug Metab Dispos.

[12] Shou M, Lin Y, Lu P, Tang C, Mei Q (2000). Enzyme kinetics of cytochrome P450 mediated reactions.. Curr Drug Metab.

[13] Atkins WM (2005). Non-Michaelis-Menten kinetics observed in CYP catalyzed reactions.. Ann Rev Pharmacol Toxicol.

[14] Koley AP, Robinson RC, Friedman FK (1996). Cytochrome P450 conformation and substrate interactions as probed by CO binding kinetics.. Biochimie.

[15] Copeland RA (2000). Enzymes: A practical introduction to structure, mechanism and and analysis: Wiley.

[16] Hollenberg PF (1992). Mechanisms of cytochrome P450 and peroxidase-catalyzed xenobiotic metabolism.. FASEB J.

[17] Guengerich FP, Yun CH, McDonald TL (1996). Evidence for a 1-electron oxidation mechanism in N-dealkylation of N,N-dialkylanilines by cytochrome P4502B1.. J Biol Chem.

[18] Zhang R, Nagraj N, Lansakara DSP, Hager LP, Newcomb M (2006). Kinetics of two-electron oxidations by the Compound I derivative of chloroperoxidase, a model for cytochrome P450 oxidants.. Org Lett.

[19] McCarthy MB, White RE (1983). Functional differences between peroxidase compound I and the cytochrome P450 reactive oxygen intermediate.. J Biol Chem.

[20] Bhakta MN, Wimalasena K (2005). A Mechanistic comparison between cytochrome P450 and chloroperoxidase catalyzed N-dealkylation of N,N-dialkyl anilines.. Eur J Org Chem.

[21] Schlichting I, Berendzen J, Chu K, Stock AM, Maves SA (2000). The catalytic pathway of cytochrome P450cam at atomic resolution.. Science.

[22] Manoj KM (2007). A novel scheme to explain the mechanistic chemistry of CYP reactions..

[23] Manoj KM (2006). Chlorinations catalyzed by chloroperoxidase occur via a diffusible intermediate and the reaction components play multiple roles in the overall process.. Biochim Biophys Acta- Proteins & Proteomics.

[24] Casella L, Poli S, Gullotti M, Selvaggini C, Beringhelli T (1994). Chloroperoxidase catalyzed oxidation of phenols: Mechanism, selectivity and characterization of enzyme-substrate complexes.. Biochemistry.

[25] Porter TD (2002). The roles of cytochrome b5 in cytochrome P450 reactions.. J Biochem Mol Toxicol.

[26] Kumar S, Davydov DR, Halpert JR (2005). Role of cytochrome b5 in modulating peroxide-supported CYP3A4 activity: Evidence for a conformational transition and cytochrome P450 heterogeneity.. Drug Metab Disp.

[27] Collom SL, Laddusaw RM, Burch AM, Kuzmic P, Perry MD (2008). CYP2E1 substrate inhibition: Mechanistic interpretation through an effector site for monocyclic compounds.. J Biol Chem.

[28] Yun CH, Yim SK, Kim DH, Ahn T (2006). Functional expression of human cytochrome P450 enzymes in *Escherichia coli*.. Curr Drug Metab.

